# Ecological Niche Characteristics of the Diets of Three Sympatric Rodents in the Meili Snow Mountain, Yunnan

**DOI:** 10.3390/ani14162392

**Published:** 2024-08-18

**Authors:** Feng Qin, Mengru Xie, Jichao Ding, Yongyuan Li, Wenyu Song

**Affiliations:** 1 Vector Laboratory, Institute of Pathogens and Vectors, Yunnan Provincial Key Laboratory for Zoonosis Control and Prevention, Dali University, Dali 671000, China; qinfeng0609@163.com (F.Q.); xiemengru1107@163.com (M.X.); liyongyuan@kmxykmsnyxxkm.wecom.work (Y.L.); 2 School of Basic Medicine, Dali University, Dali 671000, China; dingjichao1979@163.com; 3 Key Laboratory of Genetic Evolution and Animal Models & Yunnan Key Laboratory of Biodiversity and Ecological Conservation of Gaoligong Mountain, Kunming Institute of Zoology, Chinese Academy of Sciences, Kunming 650223, China

**Keywords:** biotic factor, feeding strategies, Meili Snow Mountain, metabarcoding, omnivore, small mammal, trophic niche

## Abstract

**Simple Summary:**

Investigating the dietary preferences and ecological niche characteristics of mammals can reveal their adaptive strategies under environmental changes, as well as the interrelationships and coexistence mechanisms among coexisting sympatric species. In this study, we analyzed the stomach contents of three coexisting rodents commonly found in the Meili Snow Mountain, Yunnan Province, China, using DNA metabarcoding technology to elucidate their food composition and diversity. The results revealed that, despite the presence of ecotope overlap, the three species were clearly separated in terms of their main food sources, suggesting that they may coexist in the same habitat by using different proportions and types of food to minimize interspecific food competition.

**Abstract:**

Understanding the dietary preferences and ecological niche characteristics of mammals not only reveals their adaptive strategies under environmental changes but also reveals the interspecific relationships and coexistence mechanisms among sympatric species. Nevertheless, such data are scarce for rodents inhabiting areas spanning a wide altitude range. This study employed DNA metabarcoding technology to analyze the stomach contents of *Apodemus ilex*, *Apodemus chevrieri*, and *Niviventer confucianus*, aiming to investigate their dietary compositions and diversity in the Meili Snow Mountain in Yunnan Province, China. Levins’s and Pianka’s indices were used to compare the interspecific niche breadth and niche overlaps. The results revealed the following: (1) Insecta (relative abundance: 59.4–78.4%) and Clitellata (relative abundance: 5.2–25.5%) were the primary animal food sources for the three species, while Magnoliopsida (relative abundance: 90.3–99.9%) constitutes their main plant food source. Considerable interspecific differences were detected in the relative abundance of primary animal and plant foods among the three species; (2) There was partial overlap in the genus-level animal food between *A. ilex* and *N. confucianus* (*O_jk_* = 0.4648), and partial overlap in plant food between *A. ilex* and *A. chevrieri* (*O_jk_* = 0.3418). However, no overlap exists between *A. chevrieri* and *N. confucianus*, either in animal or plant food; (3) There were no significant interspecific differences in the α-diversity of animal and plant foods among the three species. The feeding strategies and ecological niche variations of these rodents support the niche differentiation hypothesis, indicating that they have diversified in their primary food sources. This diversification may be a strategy to reduce competition and achieve long-term coexistence by adjusting the types and proportions of primary foods consumed.

## 1. Introduction

The mechanisms of species’ coexistence have always been fundamental and central topics in the animal ecology community [1,2]. Ecologists have proposed various theories and hypotheses for coexistence mechanisms, including the resource ratio hypothesis [3], the intermediate disturbance hypothesis [4], and niche differentiation theory [5,6]. Currently, the prevailing consensus among scholars posits that niche differentiation theory serves as a fundamental framework for elucidating species’ coexistence mechanisms, wherein species evade or alleviate competition through distinctiveness in their ecological niches, thereby facilitating coexistence [7,8]. Research on niche differentiation theory mainly focuses on temporal niches [9], spatial niches [10], and trophic niches [11]. Among these, the trophic niche not only reflects the nutritional requirements of a species but also indicates its trophic position and function within the ecosystem. Revealing trophic niches through research facilitates a comprehensive understanding of interspecies competition for food resources within ecosystems and sheds light on the structural characteristics of the ecosystem’s intricate food web. Food, as the fundamental substrate for animal survival and reproduction, constitutes a pivotal component of the trophic niche. Dietary analysis, serving as the fundamental basis for trophic niche investigation, vividly elucidates the nutritional requirements of organisms, thereby reflecting their ecological functionalities and trophic interdependencies [12,13,14]. Moreover, it serves as a fundamental framework for investigating the nutritional dynamics within food webs and evaluating species’ survival status and their interspecific interactions, rendering it a pivotal aspect of contemporary research on mammalian population biology and ecological studies [15,16,17].

A wide range of methodologies are employed in animal dietary analysis, encompassing direct observation of foraging behavior, laboratory-based captive feeding experiments, food residue examination, fecal analysis, isotopic analysis, and stomach content analysis [18,19,20]. With the rapid advancements in molecular biology and sequencing technologies, DNA barcoding-based molecular identification has become increasingly prevalent in animal dietary analysis [21,22,23,24]. Compared to conventional methods, DNA metabarcoding technology eliminates the reliance on traditional taxonomic expertise, thereby significantly augmenting the accuracy and precision of species identification. The technique, characterized by its exceptional resolution, efficiency, and minimal sample volume requirements, has gained increasing prominence in ecological disciplines such as biodiversity monitoring and agricultural pest management [25,26,27,28,29,30,31].

Rodents, as the most species-rich group among mammals in terrestrial ecosystems, play a pivotal role as both consumers and secondary producers. They are essential components of ecosystems, contributing significantly to energy flow and material-cycling processes [32,33]. The population density and ecological functions of rodents play a crucial role in upholding the equilibrium and stability of ecosystems, thus underscoring their significant ecological importance. Therefore, elucidating their dietary composition and foraging strategies is imperative to unveiling the mechanisms underlying their sympatric coexistence from a trophic niche perspective. This study investigated the Lantsang field mouse (*Apodemus ilex*), Chevrieri’s field mouse (*Apodemus chevrieri*), and Confucianus Niviventer (*Niviventer confucianus*). *A. ilex* and *A. chevrieri* are mouse-like species, whereas *N. confucianus* is a rat-like species. These three species exhibit similar ecological characteristics, including omnivorous diets, nocturnal behavior, and an affinity with forest habitats. They are rodent species that coexist in large populations co-occurring in the Meili Snow Mountain, located within he biodiversity hotspot of the south-west mountains of China. Their dietary composition and coexistence mechanisms remain poorly understood. This study utilized DNA metabarcoding technology to identify and compare the animal and plant food components of the three rodent species. Additionally, we investigated the overlap in their ecological niches from a trophic niche standpoint and examined the mechanisms of interspecific competition and coexistence among them.

## 2. Materials and Methods

### 2.1. Study Area

The Meili Snow Mountain is situated in Deqin County of Diqing Tibetan Autonomous Prefecture, Yunnan Province. It is located at the southeastern periphery of the Qinghai–Tibet Plateau, nestled amidst the Hengduan Mountain range on the Sino–Tibetan border between the Mekong River and Salween River. It covers an area of approximately 346 km^2^ between 28°17′ to 28°35′ N and 98°36′ to 98°52′ E. The Mekong River meanders along the eastern flank of the Meili Snow Mountain, with its riverbank reaching a minimum elevation of approximately 2000 ma.s.l. The Meili Snow Mountain exhibits an impressive elevation range of 4720 m within a horizontal distance of 30 km from the Mekong River valley to the mountain peak. This area is shaped by a combination of low-latitude climatic influences and substantial changes in elevation, resulting in a distinctive highland temperate monsoon climate characterized by year-round low temperatures and pronounced seasonality in precipitation patterns. The average annual temperature of this region is 6.02 °C [34] and the average annual rainfall measures 798.95 mm, with a distinctive rainy season lasting from early summer (May) to early autumn (September). The region boasts exceptional plant diversity encompassing nine main vegetation types, thirteen subtypes, thirty-two formations, and more than forty community types [35,36]. Its fauna includes 47 species belonging to 18 different families of wild mammals [37]. The Meili Snow Mountain’s extraordinary climate, topography, hydrology, and soil conditions foster an extensive array of plant and animal life that solidify its status as both a global biodiversity hotspot and one of China’s primary centers for endemic species’ emergence and diversification. The complex terrain and diverse plant communities within the Meili Snow Mountain region contribute to high spatial heterogeneity that supports numerous microhabitats facilitating the coexistence of multiple small mammalian species such as rodents [38].

### 2.2. Collection of Samples

Field investigations were scheduled prior to the onset of the rainy season from April to July 2023. The study consisted of five transects that were evenly dispersed throughout an altitude range of 2000–3600 m. Each transect was separated by an altitude spacing of 400 m. We employed three different sampling instruments: (1) Sherman traps (HB Sherman Traps Inc., Tallahassee, FL, USA) measuring 7.62 cm × 8.89 cm × 22.86 cm; (2) museum snaps measuring 10 cm × 6 cm; and (3) buckets measuring Φ20 cm × 20 cm. The total number of placements for all three tools combined per transect is around 1000. A group of traps consisting of one Sherman trap and one snap trap, with each trap placed 1–2 m apart, was established. Within the transect, the researchers observed the walking trails of small mammals and dug holes along these trails to position bucket traps. The transects were divided into two sub-transects. Traps were placed in the first sub-transects of the first transect on the first day. On the second day, the traps were replenished with bait. On the third day, the traps were moved to the second sub-transects of the first transect. The same work conducted on the second day was repeated on the fourth day. On the fifth day, the traps were moved to the first sub-transects of the second transect. This sampling process was repeated in each transect until sampling was completed in all transects [39]. The captured animals were initially identified in the field based on external characteristics. Muscle tissue was extracted from each specimen and preserved in 99.7% ethanol for storage in a laboratory freezer at −80 °C. Within each altitude segment, five representative samples for every species were selected based on morphological and molecular identification. For molecular identification, the total DNA from the tissue samples was extracted using an animal tissue DNA extraction kit (TIANGEN Biochemical Technology Co., Ltd., Beijing, China). Species were identified through DNA barcoding based on the *Cytb* gene [40]. The samples were sent to Tsingke Biotechnology Co., Ltd. (Kunming, China) for bidirectional sequencing. The sequencing results were analyzed using the BLAST algorithm in the publicly available NCBI database (https://www.ncbi.nlm.nih.gov/ accessed on 17 January 2024) to retrieve species-specific information. Mixed stomach content samples were collected from the same species within specific altitude segments (*Apodemus chevrieri* was absent at 2400 m, and *N. confucianus* was absent at 3600 m), followed by DNA extraction from the stomach chyme. A total of 13 chyme DNA samples were utilized for subsequent sequencing after excluding segments without captured samples.

### 2.3. Sampling of Gut Contents, DNA Extraction and Sequencing

The DNA extraction from the chyme in the stomach contents was performed using the OMEGA Soil DNA Kit (M5635-02) (Omega Bio-Tek, Norcross, GA, USA). To identify animal-derived food, the mitochondrial *COI* (Mitochondrially Encoded Cytochrome C Oxidase I) gene was utilized, while the *RbcL* (ribulose-bisphosphate carboxylase gene) was employed for plant-derived food identification [41]. The primers used were COIZBJ-ArtF1c (AGA TAT TGG AAC WTT ATA TTT TAT TTT TGG)/COIZBJ-ArtR2c (WAC TAA TCA ATT WCC AAA TCC TCC) for *COI* and Z1aF (ATG TCA CCA CCA ACA GAG ACT AAA GC)/hp2R(CGT CCT TTG TAA CGA TCA AG) for *RbcL* [42,43]. The amplified products were quantified using Nanodrop and subsequently subjected to electrophoresis in 1.2% agarose gel for verification purposes. Those that satisfied the amplification and verification criteria were sequenced on the Illumina NovaSeq-PE250 platform at Shanghai Personal Biotechnology Co., Ltd. (Shanghai, China). with a read length of 250 bp for each end.

### 2.4. Sequence Processing and Data Statistical Analysis

The raw sequencing data were initially processed to remove primers and adapters using cutadapt (v2.3); sequences were then assembled, quality-controlled, deduplicated, and chimera-checked using Vsearch (v2.13.4_linux_x86_64), resulting in high-quality sequences. These high-quality sequences were clustered at a 97% similarity level using the “cluster_size module” [44]. The representative sequences of operational taxonomic units (OTUs) were annotated at various taxonomic ranks, including kingdom, phylum, class, order, family, genus, and species. This annotation was performed using the QIIME2 software (version 2019.4) in conjunction with the NCBI database [45,46]. The sequence alignment results exhibit a minimum consistency of 99%, with only one species corresponding to the most matched sequence. In cases where this species is locally distributed, the sequence is considered to originate from that specific species. On the other hand, if the sequence alignment consistency exceeds 98%, it can be annotated at the genus level. The taxonomic composition was visualized using QIIME2 (2019.4), where the qiime taxa barplot module was employed. To depict the taxonomic hierarchy based on abundance data grouped by each OTU, treemapping techniques including a circle packing diagram were implemented in R 4.3.2 with the ggraph and ggplot2 packages [47,48]. The dietary α-diversity indices were calculated using the Chao1 estimator, Simpson’s diversity index, and Shannon–Wiener diversity index [49,50] and analyzed using the QIIME2 software. The dietary β-diversity values of the samples were determined by conducting a nonmetric multidimensional scaling analysis (NMDS) using the Jaccard distance matrix [51]. In NMDS clustering findings, the distance between the points is directly proportional to the level of difference exhibited by the points in the original data. The stress value quantifies the degree of fit between the NMDS configuration and the original distance matrix. As stress decreases, the NMDS configuration aligns more closely with the original data. A stress number below 0.1 indicates a favorable outcome, with no chance of obtaining misleading information [52]. Data visualization was performed using the ggplot2 package in R v.4.3.2, while intergroup differences were assessed for significance using the Analysis of Similarities (ANOSIM) method. After performing Kruskal–Wallis and Wilcoxon tests, we employed an LEfSe (Linear Discriminant Analysis Effect Size) analysis as described by Segata [53] to identify taxonomic groups exhibiting differential abundance across the three species under investigation. Our focus was on identifying groups with a *p*-value lower than 0.05 and an LDA score exceeding 2 at various classification levels [53]. To compare the significant differences in food composition and diversity among different species, this study employed one-way analysis of variance (ANOVA) and Student’s *t*-test in SPSS 26 to identify statistically significant disparities.

### 2.5. Characteristics of Trophic Niche

The trophic niche characteristics were calculated in this study using the following formulas. The Levins index (*B*) was applied to quantify the diet niche breadth [54]. The Pianka overlap index (*O_jk_*) was employed to assess the magnitudes of overlap of the dietary niche [55].

$$B=\frac{1}{\sum P_{i}^{2}}$$

where *B* represents the niche breadth, and *P_i_* represents the proportion of food item *i*.

$$O_{jk}=\frac{\sum \left(P_{ij}\cdot P_{ik}\right)}{\sqrt{\sum (P_{ij}^{2})\cdot \sum \left(P_{ik}^{2}\right)}}$$

where *P_ij_* and *P_ik_* represent the relative abundance of food item *i* in the diets of species *j* and *k*, respectively. The value of *O_jk_* ranges between 0 and 1, with a higher value indicating a greater degree of dietary overlap. A threshold of *O_jk_* > 0.3 is considered to indicate meaningful overlap, while *O_jk_* > 0.6 is regarded as indicative of significant overlap [56].

## 3. Results

### 3.1. Food Composition of Studied Species

**Figure 1:**
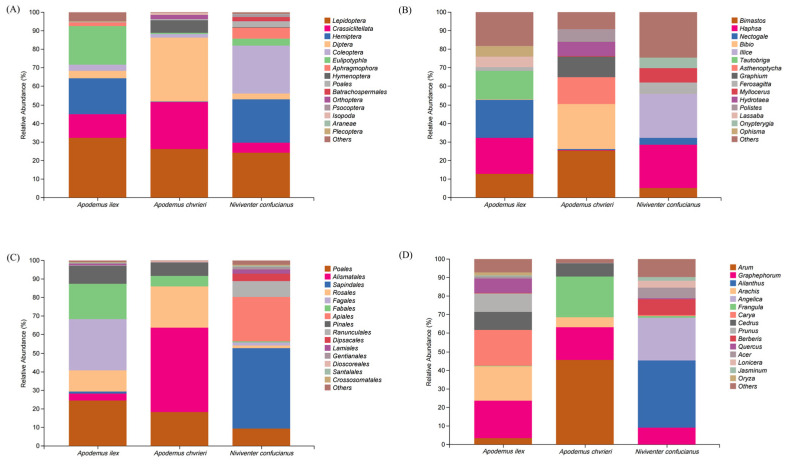
Relative abundance (RA) of the top 15 food items for three species. The components of animal-based food are classified at the (**A**) order and (**B**) genus levels, and the components of plant-based food are classified at the (**C**) order and (**D**) genus levels.

A total of 1,800,078 sequences were obtained from the stomach content samples of three species using COI metabarcode sequencing, with an average of 138,467 ± 3978 sequences per sample. Analyzing the stomach contents of the three rodent species could reveal the following conclusions regarding their animal dietary sources. At the order level, *A. ilex* primarily consumed Lepidoptera (relative abundance [RA] = 32.26%), Eulipotyphla (RA = 20.57%), Hemiptera (RA = 19.29%), and Crassiclitellata (RA = 12.92%). *Apodemus chevrieri* mainly fed on Diptera (RA = 34.43%), Lepidoptera (RA = 26.15%), and Crassiclitellata (RA = 25.51%). *Niviventer confucianus* mostly fed on Coleoptera (RA = 25.93%), Lepidoptera (RA = 24.39%), and Hemiptera (RA = 23.55%). At the taxonomic level of genus, the diet of *A. ilex* consisted mainly of *Nectogale* (RA = 20.57%) belonging to the Eulipotyphla order, *Haphsa* (RA = 19.26%) belonging to the Hemiptera order, *Tautobriga* (RA = 15.34%) belonging to the Lepidoptera order, and *Bimastos* (RA = 12.92%) belonging to the Crassiclitellata order. The diet of *A. chevrieri* mainly includes *Bimastos* (RA = 25.51%) belonging to the Crassiclitellata order, *Bibio* (RA = 24.11%) belonging to the Diptera order, *Asthenoptycha* (RA = 14.62%) and *Graphium* (RA = 11.1%) belonging to the Lepidoptera order. The diet of *N. confucianus* mainly consists of *Haphsa* (RA = 23.44%) belonging to Hemiptera order and *Illice* (RA = 23.42%) belonging to Lepidoptera order. (Figure 1A,B).

Based on the RbcL metabarcode identification data for plant-based food, a total of 1,247,647 valid sequences were obtained through high-throughput sequencing of all samples, with an average of 95,973 ± 17,516 sequences per sample. The plant-based food consumed by all three species predominantly belongs to the class Magnoliopsida (dicotyledons), exhibiting a relative abundance exceeding 90% for each species. At the order level, *A. ilex* mainly devoured Fagales (RA = 27.62%), Poales (RA = 24.66%), Fabales (RA = 18.95%), and Rosales (RA = 11.58%). *Apodemus chevrieri* primarily consumed Alismatales (RA = 45.62%), Rosales (RA = 22.16%), and Poales (RA = 18.18%). *Niviventer confucianus* mainly fed on Sapindales (RA = 43.15%) and Apiales (RA = 23.91%). At the genus level, *A. ilex* mostly consumed *Graphephorum* (RA = 20.06%) from the Poales order, *Carya* (RA = 19.47%) from the Fagales order, *Arachis* (RA = 18.56%) from the Fabales order, and *Prunus* (RA = 10.08%) from the Rosales order. The diet of *A. chevrieri* consisted mainly of *Arum* (RA = 45.48%) from the Alismatales order, *Frangula* (RA = 21.76%) from the Rosales order, and *Graphephorum* (RA = 17.84%) from the Poales order. The diet of *N. confucianus* mainly consists of *Ailanthus* (RA = 36.12%) from the Sapindales and *Angelica* (RA = 22.95%) from the Apiales order, as depicted in Figure 1C,D.

**Figure 2:**
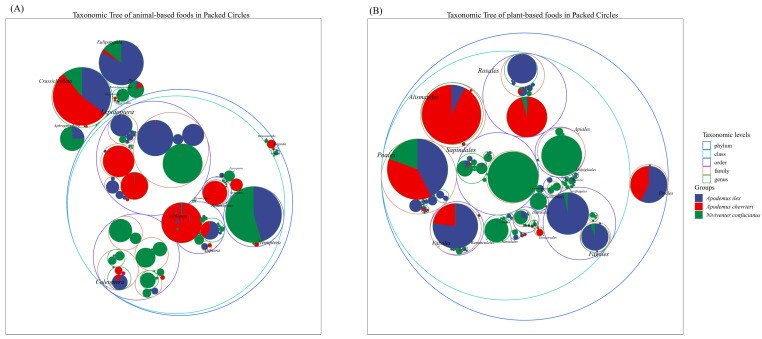
Taxonomic hierarchy tree of the dietary composition of the three species. (**A**) Animal-based foods; and (**B**) plant-based foods.

The Insecta class of the phylum Arthropoda constitutes the primary source of animal sustenance for the three species. However, notable disparities exist in their dietary preferences, with minimal overlap observed in their alimentary spectra. For example, within the Lepidoptera order, *A. ilex* predominantly exhibits a preference for members of the Geometridae and Erebidae families, particularly genera such as *Tautobriga* and *Ophisma*. *Niviventer confucianus* demonstrates a specific inclination towards the genus *Illice* within the Erebidae family. In contrast, *A. chevrieri* incorporates dietary components derived from the Tortricidae and Papilionidae families. *Niviventer confucianus* exhibits a preference for preying on insects from the Coleoptera order, including those from the families Curculionidae, Carabidae, Chrysomelidae, and Cantharidae, which are present in its diet. In contrast, *A. ilex* and *A. chevrieri* have only been found to contain negligible amounts of Coleoptera in their stomach contents. Hemiptera are preyed upon by *A. ilex* and *N. confucianus*, while no Hemiptera components were detected in the stomach of *A. chevrieri*. In addition to the class Insecta, Eulipotyphla species serve as a preferred food source for *A. ilex*, are consumed in smaller quantities by *N. confucianus* and are found in limited amounts in *A. chevrieri*. Species from the order Crassiclitellata, on the other hand, constitute a common food source for all three species (Figure 2A).

In terms of the plant-based diet, *A. ilex* and *A. chevrieri* exhibit greater dietary similarities, as both species primarily consume plants belonging to the orders Poales, Fabales, Alismatales, and Pinales. As a consequence, there is a partial overlap in their feeding habits. Within the order Rosales, *A. ilex* primarily consumes plant species belonging to the family Rosaceae, while *A. chevrieri* predominantly includes components from the family Rhamnaceae in its diet. The dietary preference of *N. confucianus* is concentrated on plant species from the orders Sapindales, Ranunculales, and Apiales, with only trace amounts detected in samples from *A. ilex* and *A. chevrieri.* Furthermore, the order Fagales represents a favored group of plants consumed by *A. ilex*, whereas *N. confucianus* also consumes it but in a lesser proportion (Figure 2B).

### 3.2. Interspecific Comparison of Food Composition Diversity

**Figure 3:**
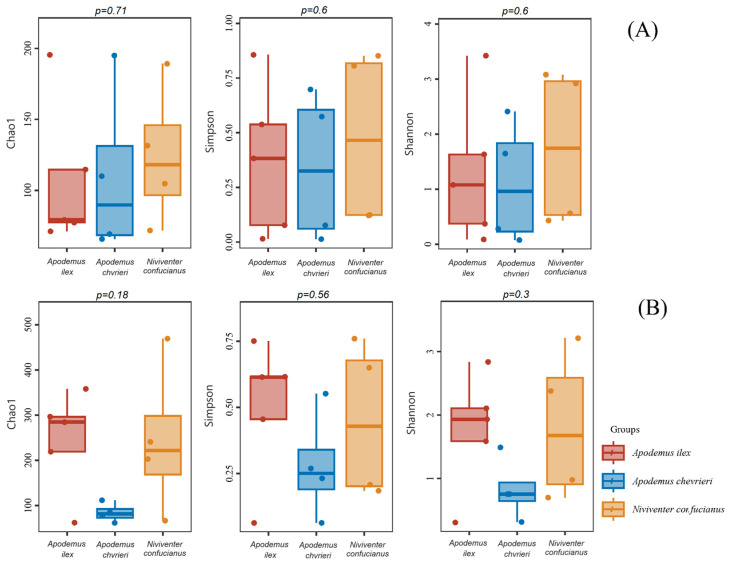
Box-and-whisker plots for α-diversity of the diet compositions of the three species. The *X*-axis shows sample type. The *Y*-axis shows the value of each diversity. The *p*-value for each diversity index according to the Kruskal–Wallis test is located above the box-and-whisker plots. (**A**) Animal-based foods; and (**B**) plant-based foods.

The α-diversity estimates indicated that there were no statistically significant differences observed in the dietary diversity of the three species, regardless of animal- or plant-based food sources (Figure 3).

**Figure 4:**
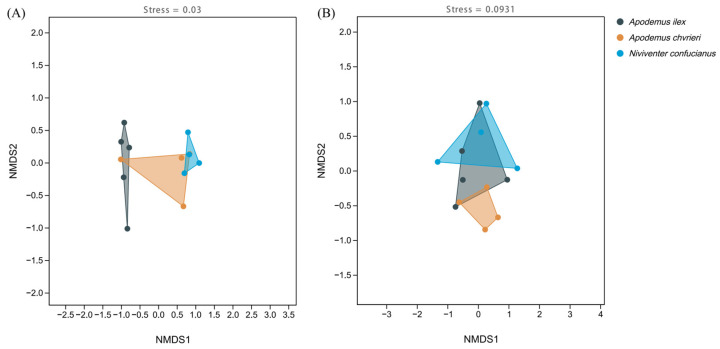
Non-metric multidimensional scaling (NMDS) of the dietary composition of the three species based on Jaccard distance. (**A**) Animal-based foods; and (**B**) plant-based foods.

The NMDS clustering results illustrate the interspecific variations in the composition of animal and plant foods among these species (Figure 4). All stress levels are below 0.1, indicating that the results are reliable. Additionally, when looking at the distances between the dots on the graph, it becomes evident that there is greater variation in the animal food sources among the three rodent species, but there is more similarity in the concentrated content of the plant meals. The clustering findings demonstrate the rivalry among the three species for food supplies and their utilization of distinct foraging tactics to acquire a wider variety of food, hence minimizing competition.

### 3.3. Difference Analysis of Food between the Three Species

**Figure 5:**
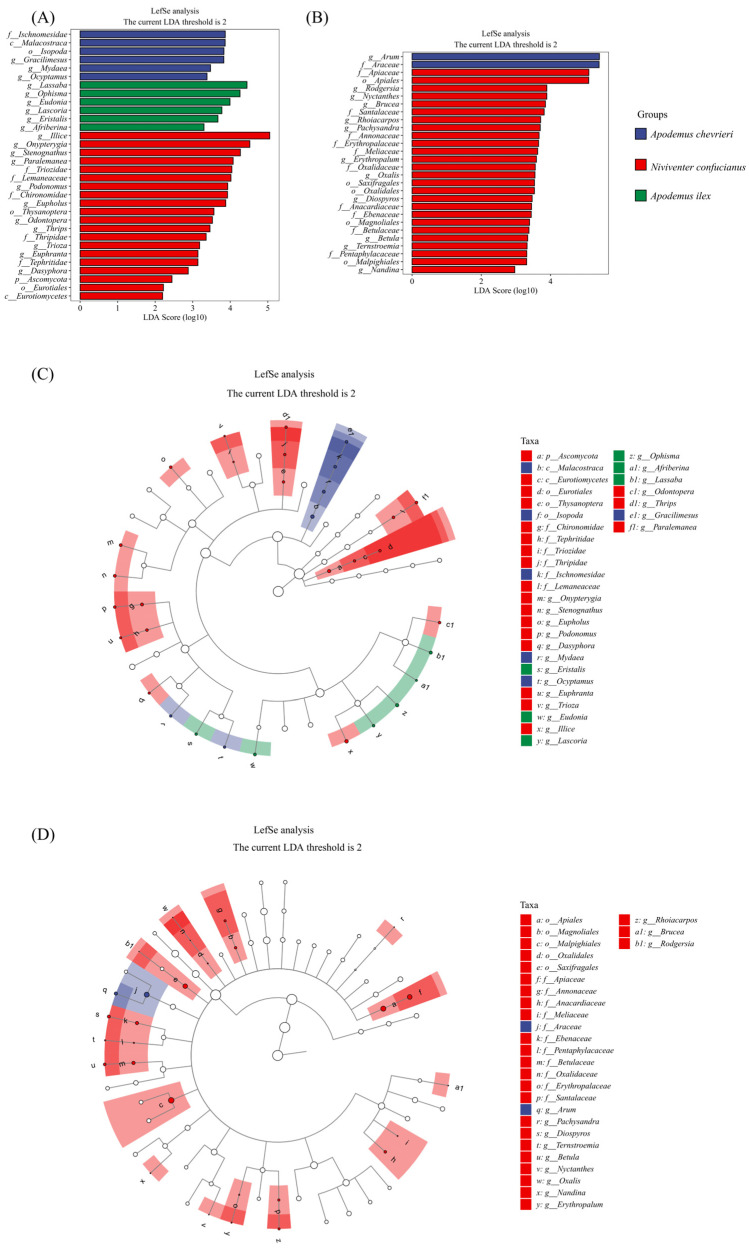
LEfSe analysis identified biomarkers in the food components of the three species. LDA scores above 2 and *p* values smaller than 0.05 were shown. (**A**,**B**) The histogram of Linear Discriminant Analysis (LDA) scores displays distinct species (biomarkers) with significantly different abundances across the three species. The length of each bar, representing the LDA score, indicates the effect size, which is the degree to which a biomarker accounts for the phenotypic variation among the groups. (**C**,**D**) The cladogram depicts taxonomic levels from phylum to genus (species) through concentric circles, with each circle’s diameter reflecting the taxon’s relative abundance. (**A**,**C**) Animal-based foods; and (**B**,**D**) plant-based foods.

Applying a thorough LEfSe analysis, we have precisely identified the important differences in food choice across three rodent species (Figure 5A,C). Within the field of animal-based nourishment, *A. chevrieri* has a clear preference for prey belonging to the class Malacostraca, specifically narrowing down its choices to the order Isopoda. At the familial level, it exhibits a predilection for creatures belonging to the order Ischnomesidae. At the genus level, the variety is evident, with *Gracilimesus*, *Mydaea*, and *Ocyptamus* being particularly prominent. The *A. ilex* has a pronounced preference for certain genera, namely *Lassaba*, *Ophisma*, and *Eudonia*. Meanwhile, *N. confucianus* consumes a wide variety of food items, particularly showing a strong preference for the order Thysanoptera and the families Triozidae, Lemaneaceae, and Chironomidae. At the genus level, it specifically targets *Illice*, *Onypterygia*, and *Stenognathus* as its primary food sources.

In plant-based foods, *A. chevrieri* leans towards specific species within the families Arum and Araceae as its quintessential sources of nourishment. *Niviventer confucianus*’s plant diet specificity is even more diverse, selecting plants from five orders, nine families, and twelve genera. Meanwhile, *A. ilex* displays a broader dietary pattern, without exhibiting the same level of specificity in its plant choices (Figure 5B,D).

### 3.4. Trophic Niche Breadth and Overlap Index

In the assessment of genus-level trophic niche width for the three species, it was observed that both *A. ilex* (B = 7.6388) and *N. confucianus* (B = 7.5649) exhibit comparable trophic niche widths for animal-based foods, surpassing that of *A. chevrieri* (B = 5.9610). Concerning plant-based foods, *A. ilex* demonstrates the widest trophic niche width (B = 7.1344), followed by *N. confucianus* (B = 4.8972), while *A. chevrieri* exhibits the narrowest value (B = 3.3981) (Table 1).

Pianka’s index yielded the highest niche overlap between *A. ilex* and *N. confucianus* (*O_jk_* = 0.4648) among the three species at the genus level for animal-based foods (Table 2). This finding suggests potential competition for animal-based food resources between these two species. Contrastingly, *A. chevrieri* exhibited no overlap with either other species in terms of animal-based food resources, indicating a lack of competitive relationships. Regarding plant-based trophic ecology, there was a moderate degree of overlap between *A. ilex* and *A. chevrieri,* with an *O_jk_* value of 0.3418 (Table 2), while the overlap indices between *N. confucianus* and the other two species were not statistically meaningful (*O_jk_* < 0.3).

## 4. Discussion

### 4.1. Dietary Differences among the Three Species

Using DNA metabarcoding technology, we examined the dietary composition during non-rainy seasons and interspecific competition for food resources among three sympatric species in the Meili Snow Mountain, Yunnan. The findings indicate that the orders Lepidoptera, Crassiclitellata, Hemiptera, Dipteran, Coleoptera, and Eulipotyphla constitute the primary animal food sources for the three species. A study examining the dietary preferences of birds revealed that insects, including Lepidoptera and spiders, offer a higher concentration of protein, fat, and trace components. Consequently, birds exhibit a greater inclination towards selecting these nutrient-dense insects [57]. The findings of the present study indicate that there was some overlap between the results obtained and those of that previous study. This suggests that the animal-based elements of the diets of all three rodent species had greater quantities of Lepidopteran insects due to their superior nutritional value. Regarding plant-based nutrition, their diets predominantly comprised species of the orders Poales, Alismatales, Sapindales, Rosales, Fagales, and Apiales. Despite the overlaps in trophic niches among the different species, a clear segregation is observed in their primary food sources, indicating that they potentially mitigate interspecific competition for food resources by consuming distinct proportions and types of food, thereby facilitating coexistence within the same habitat.

The main animal-based diet of the *A. ilex* includes the orders Lepidoptera, Crassiclitellata, Hemiptera, and Eulipotyphla. The plant-based food predominantly includes the orders Poales, Rosales, Fagales, and Fabales. The dietary preferences of *A. ilex* indicate that some species, such as Crassiclitellata and Poales, which inhabit grassy regions, share habitats with *A. ilex* in shrubby and weedy environments. This overlap facilitates *A. ilex*’s access to these species, making it simpler to consume them. Nevertheless, certain species, including Lepidoptera and Fagales, have different habitats, indicating that *A. ilex* is extending its geographical range to obtain more sustenance for survival [58]. Due to the reclassification of *A. ilex* as a distinct species [59], limited ecological information is available on its dietary habits. Our research presents a pioneering account of the dietary habits of *A. ilex*. Furthermore, it is particularly intriguing that our findings have revealed small mammals (Eulipotyphla) to be constituents of *A. ilex’s* diet.

*Apodemus chevrieri* is endemic to China. In Xichang and Sichuan, *A. chevrieri* exhibits a preference for a seed-based diet, as evidenced by the presence of starch-rich foods in its stomach, while the occurrence of green chyle is infrequent and no trace of animal-based foods has been observed [60]. The molars of *A. chevrieri* imply a dietary preference for rice, corn, grass seeds, and tree seeds, thereby leading to the notion that it is an exclusive herbivore [61]. Nevertheless, a decade-long continuous study on the biological characteristics of *A. chevrieri* in Yanyuan, Sichuan, revealed that it is omnivorous. The examination of stomach contents has revealed that, from December to March, its diet primarily comprises grass roots and plant seeds, while from April to November it predominantly includes grass stems, plant seeds, and insect remnants [62]. Chen et al. [63] conducted a study on the dietary composition of four rodent species inhabiting the Shennongjia area and revealed that *A. chevrieri* primarily consumes seeds (42.9%) and animal-derived food (57.1%). We detected both plant-based and animal-based food components in the diet of *A. chevrieri*, which may be attributed to our capture period from May to July, corresponding to the pre-rainy season in the Hengduan Mountains. The samples from *A. chevrieri* demonstrated that the plant-based components of its diet primarily comprise the orders Poales, Alismatales, and the Rosales taxa. The majority of these plants possess starch-rich tubers or fruits, which aligns with the findings from microscopic examination of *A. chevrieri*’s stomach contents [60,62,63]. The present study’s findings exhibit notable resemblances to those of prior research in terms of plant-based dietary constituents, while demonstrating enhanced precision in specific components. In terms of animal-based food, the findings of our study further support the conclusion that *A. chevrieri* exhibits dietary preferences for both plant matter and animal-based food across different seasons, thus confirming its classification as an omnivorous species.

Previous research findings indicate that *N. confucianus* exhibits a diverse dietary profile, displaying omnivorous behavior with a predominant consumption of starch-based foods. Their diet composition varies seasonally, with an increased proportion of carnivorous food in spring, greater intake of berries during summer and autumn, and a primary reliance on starch-rich foods in winter [63,64]. This study provides a more detailed characterization of both animal and plant components in the diet of *N. confucianus* compared to the existing literature. A study on six small mammal species in the Laojunshan National Nature Reserve in Sichuan revealed that *N. confucianus* frequently consumes the moth genus *Uropyia* [24]. The animal diet of *N. confucianus* in this study predominantly comprises the animal orders Lepidoptera, Hemiptera, and Coleoptera, while their plant diet primarily consists of the families Sapindales and Apiales. *N. confucianus* possesses a greater physical size in comparison to *A. ilex* and *A. chevrieri* [65]. This bigger body size enables *N. confucianus* to occupy a broader habitat and have easier access to insects. Therefore, analysis of its dietary preferences indicates that a significant portion of its meal consists of the class Insecta.

The observed disparities compared to previous studies can be attributed to variations in habitat types at the sampling locations as well as the spatial distribution of food resources. Additionally, variations in the life history characteristics of insects (e.g., their developmental stages and sizes) may exert an influence on the prey choices and predation efficiency of small mammals, potentially leading to discrepancies in dominant food group composition across different studies [66]. Additionally, the sampling time in this study only allowed for recording the dietary composition of the three species before the rainy season. It is possible that seasonal variations could impact interspecific competition by altering dietary composition, which may offer new avenues for future research.

### 4.2. Trophic Niche Characteristics of the Three Species

Prey type has been suggested to be an important aspect of macroevolution and macroecology in mammals [67]. Analyzing food composition can also help to predict the ways population dynamics and individual behaviors influence the fitness of target species [68,69,70]. The trophic niche reflects an organism’s resource utilization and its capacity to adapt to habitats. Trophic niche breadth indicates an animal’s nutritional level and ecological relationships, while trophic niche overlap can effectively serve as an indicator of resource utilization competition among sympatric species [71]. For example, a study of bats across a 1500 m elevational gradient in the Himalayas indicated that coexisting species in the low elevation regions occupied a small niche width with high overlap (niche packing), whereas species in the high elevation areas showed large niche width with low overlap (niche partitioning) [72]. Such variations in dietary niche structure were found to influence food webs through coexistence mechanisms using different strategies among the species [73], further improving our knowledge and contributing towards the more efficient management of ecosystem functioning [73,74].

Among the three species examined in this study, *A. ilex* exhibited a remarkably high level of both animal and plant food niche breadth (Table 1), indicating its pronounced proficiency in utilizing food resources and adapting to diverse habitats. The animal food niche breadth of *A. chevrieri* is slightly smaller compared to that of *A. ilex* and *N. confucianus*, but the difference is not statistically significant. However, it exhibits the narrowest trophic niche breadth for plant foods (Table 1), indicating a stronger selectivity towards a plant-based diet. The plant-based diet of *A. chevrieri* exhibited a high RA (45.5%) of the species *Arum dioscoridis*, which could potentially contribute to its limited trophic niche breadth in terms of plant food consumption.

Our findings demonstrate a partial overlap in the consumption of animal-based food resources between *A. ilex* and *N. confucianus*, while also revealing a partial overlap in plant-based food resources between *A. ilex* and *A. chevrieri*. There is minimal dietary overlap observed between *A. chevrieri* and *N. confucianus*. The Meili Snow Mountain is characterized by rugged topography and fast-flowing rivers. This area harbors some of the most intricate river systems, mountain ranges, and geological history in the world, which may support a large trophic niche breadth for each species and a low degree of dietary overlap, such as those reported in bats [23]. The exceptional natural geographical conditions in this area make it one of China’s and the world’s most resource-rich regions. As an endemic and dominant species in this region, *A. ilex* exhibits partial overlap with both *A. chevrieri* and *N. confucianus* in animal-based and plant-based foods, indicating interspecific competition for resources. However, there is discernible differentiation in their dietary preferences and a clear separation in their trophic niches. Therefore, the segregation of trophic niches plays a crucial role in facilitating the sympatric coexistence of these three species.

## 5. Conclusions

Our findings reveal discernible disparities in the feeding preferences for primary food sources among the three species, implying their potential utilization of divergent foraging strategies to achieve distinct food proportions. This adaptive approach effectively mitigates competition for limited food resources within their habitat, thereby facilitating long-term coexistence. Moreover, comprehending rodents’ dietary preferences is pivotal for designing effective control and management strategies.

## Figures and Tables

**Table 1:** Trophic niche breadth of the three species.

	Animal-Derived Food		Plant-Derived Food	
	Order Level	Genus Level	Order Level	Genus Level
*Apodemus ilex*	4.8608	7.6388	5.0680	7.1344
*Apodemus chevrieri*	3.8784	5.9160	3.3481	3.3981
*Niviventer confucianus*	5.1911	7.5649	3.8118	4.8972

**Table 2:** Trophic niche overlap index (*O_jk_*) of the animal- derived food items at the genus level in the three species. Bold values denote either meaningful or significant overlap.

	Animal-Derived Food		Plant-Derived Food	
	*Apodemus chevrieri*	*Apodemus chevrieri*	*Apodemus chevrieri*	*Apodemus chevrieri*
*Apodemus chevrieri*	0.2429		**0.3418**	
*Niviventer confucianus*	**0.4648**	0.0973	0.1249	0.0762

## Data Availability

The experimental data used to support the findings of this study are available from the corresponding author on request.
